# Neuroepidemiology study of headache in the region of Jammu of north Indian population: A cross-sectional study

**DOI:** 10.3389/fneur.2022.1030940

**Published:** 2023-01-05

**Authors:** Amrit Sudershan, Agar Chander Pushap, Mohd Younis, Srishty Sudershan, Sheetal Bhagat, Hardeep Kumar, Rakesh K. Panjalyia, Parvinder Kumar

**Affiliations:** ^1^Institute of Human Genetics, University of Jammu, Jammu, Jammu and Kashmir, India; ^2^Department of Human Genetics, Sri Pratap College Srinagar, Cluster University Srinagar, Srinagar, Jammu and Kashmir, India; ^3^Department of Education, University Wing, Dakshina Bharat Hindi Prachar Sabha, Chennai, India; ^4^Department of Zoology, University of Jammu, Jammu, Jammu and Kashmir, India; ^5^Department of Psychology, University of Jammu, Jammu, Jammu and Kashmir, India; ^6^Department of Neurology, Super Speciality Hospital, GMC, Jammu, Jammu and Kashmir, India

**Keywords:** headache, prevalence, Jammu, north India, migraine, tension type headache

## Abstract

**Background:**

Headache disorders now represent a major public health problem globally. It is more prevalent in developing countries with the rising trends of headache disorders observed in young adults affecting their quality of life negatively. Very little information is available on the epidemiology of headache disorders in the Jammu Division of the north Indian population.

**Aim:**

The aim of the present study was to find out the prevalence of headache and its two major types, i.e., migraine and tension-type headache (TTH), in the population of the Jammu Division.

**Methods:**

The present study was conducted in two phases: (Phase I: face-to-face interview and Phase II: E-based sampling) and the sufferers of headaches were incorporated into the study based on the International Classification of Headache Disorder-3 (ICHD-3) criteria for a representative sample. Frequency distribution and mean ± standard deviation were used in descriptive statistics to describe the data sets, while a *t*-test, chi-square test, multiple logistic regression, and prevalence ratio were used in inferential statistics.

**Results:**

In the present study, a total of 3,148 patients were recruited, with an overall prevalence of headache of 53.84%, with a majority of females (38.18%) over males (15.66%). As regards the type of headache, migraine was found to be of the more prevalent (33.25%) type than the TTH (20.58%). Females suffering from migraine showed the highest prevalence (25.28%), in contrast to females suffering from the TTH (12.89%). Sociodemographic variables, such as gender [female; AOR = 2.46, 95% CI (2.12–2.85), *p*-value < 0.0001] and marital status [married; AOR: 1.46, 95% CI (1.11–1.92) *p*-value = 0.006], showed a significant association with the headache.

**Conclusion:**

The present study shows that the prevalence of headache is high in the Jammu Division of Jammu and Kashmir (J&K) India, with migraine being the highly prevalent type.

## Graphical Abstract

Graphical abstract 
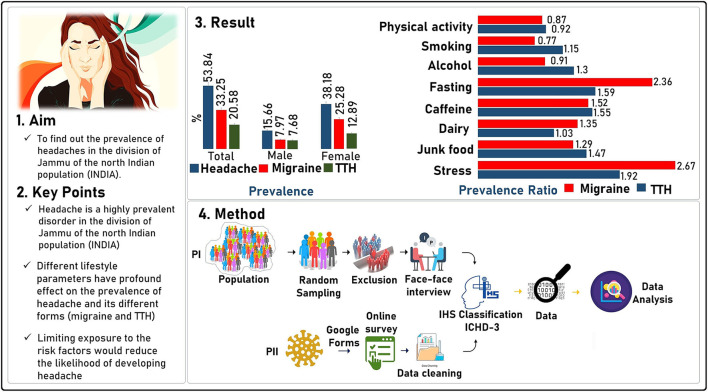

## 1. Introduction

Headache is the most common, painful, expensive, and stressful condition in the world and it is mentioned as the third topmost disabling disease after low back pain and depressive disorder (1). According to the Global Burden of Disease research-2019 (GBD-2019), headache disorders were the third most frequent cause of disability out of 369 diseases and injuries (2). Migraine and tension-type headaches (TTH) are the two most prevalent neurological disorders associated with primary headaches. The GBD-2019 has shown that the prevalence of headaches is high across different countries of the world. For instance, Italy shows the maximum frequency with 49.02% of prevalent cases per year, followed by Norway (47.98%) and Belgium (47.64%). Different developed nations such as the United States of America (the USA), Russia, the United Kingdom (the UK), and Germany also show high prevalence rates of about 42,780.87, 40,971.76, 42,509.08, and 43,855.96 prevalent cases per 100,000, respectively [GBD Compare | IHME Viz Hub healthdata.org].

Headache covers a significant portion of the global public health issues ranging from impeding everyday functioning, causing loss of productivity, increasing financial burden, to restricting social contact (3). The adolescents have higher rates of all types of headaches than younger children, which negatively impact their school activities, future life, and even family life (4). It has been estimated that only migraine considerably causes low efficiency in job productivity with greater absenteeism and less presenteeism, impairment in daily activities as well as several visits to healthcare providers. All these reasons were significantly correlated with the cause of a higher economic loss (5–7). It has been estimated that presenteeism-alone costs are estimated to be over US$1,296, which are higher than the absenteeism costs (US$370) (6).

Although the prevalence of headache is an important epidemiologic measure, understanding the prevalence and risk factors associated with headache in populous nations like India is essential to comprehend the entire scope of burden caused by headache. This development may bring about a positive impact on the prevention of any diseases that are likely to affect mankind in the future and drawing up of health promotion programs as well as present national or local initiatives which are of immense benefit to all those associated with the healthcare system. Some studies have been conducted in different regions of India, including south India, eastern India (8, 9), and the Kashmir division of Jammu & Kashmir [Union territory (UT)] (10, 11). But, due to lack of epidemiology studies on headaches in the Jammu Division (north Indian population), it is very difficult to determine the factors of how common issues pertaining to headache are prevalent in this population.

Therefore, the current study aimed to find out the prevalence of the headache condition and its two major types, including migraine and TTH, in the region of the Jammu Division of the north Indian population. To the best of our knowledge, this is the first study of its kind on neuroepidemiology to discuss headache and its types other than the case-control design (12) in the present region utilizing the cross-sectional study design; hence, this insight shall provide the foundation for future research.

## 2. Materials and methods

### 2.1. Sample selection

In the present study, utilizing the cross-sectional epidemiological study design, the subjects (aged from ≥13 to 75 years) were enrolled using a simple random sampling method from the Jammu Division of the north Indian population, from February 2021 to April 2022. The sampling process was executed in two phases, where in Phase I, the subjects were enrolled from the Jammu population by a face-to-face interview, using the simple random sampling approach. Each participant was initially briefed about the methodology and purpose of the present study and asked to provide their informed permission and consent (or guardian where applicable) before the interview was held. This interview was then conducted by the neurologist and professional interviewers. After gathering all the demographic data, a screening question was put forth to the participants: “Have you had a headache within the past year?” if he/she answered positively, only then the diagnosise, which was based on the criteria of the International Classification of Headache Disorders (ICHD-3), was done (Table 1). The diagnosis of different forms of primary headaches, such as migraine, TTH, and menstrually related migraine (MM), was carried out utilizing the ICHD-3 criteria (Table 1).

**Table 1:** Inclusion criteria for migraine and tension-type headache 10.

**Migraine**	**Tension-type headache (TTH)**	**Menstrually-related migraine (MM)**
At least five attacks fulfilling criteria A–C A. Headache attacks lasting 4–72 h (untreated or unsuccessfully treated) B. Headache has at least two of the following four characteristics: 1. Unilateral location 2. Pulsating quality 3. Moderate or severe pain intensity 4. Aggravation by or causing avoidance of routine physical activity (e.g., walking or climbing stairs) C. During headache at least one of the following: 1. Nausea and/or vomiting 2. Photophobia and phonophobia	A. Headache has at least three of the following four characteristics: 1. Bilateral location 2. Pressing/tightening (non-pulsating) quality 3. Mild or moderate intensity 4. Not aggravated by routine physical activity such as walking or climbing stairs B. No nausea, vomiting, photophobia or phonophobia	Attacks, in a menstruating woman, fulfilling criteria for Migraine and criterion A given below A. Occurring on day 1 ± 2 (i.e., days −2 to +3)^2^ of menstruation1 in at least two out of three menstrual cycles, and additionally at other times of the cycle3

But due to the coronavirus disease (COVID-19) pandemic and lockdown measures enforced during the sampling period, we had to use the E-based sampling *via* email with the aid of a Google form (Phase II). The Google form was designed strategically and systematically, into different sections, where in Section I, complete information was given to the participant about the survey and the participant in turn was asked to give their consent for the usage of their data. If an individual was willing to give their consent for the purpose of data usage only, then he/she would be able to enter into Section II where different demographic questions were already entered [such as age, weight, height, gender, occupation, address (district)], and if not, the form will automatically end up. In Section III, the headache screening question “have you had a headache during the last year,” was placed first If an individual positively accepts the question, only then would he/she be allowed to enter into the section of ICHD-3-based diagnostic criteria (Section III), and if not, the individual will automatically bypass the diagnostic section and entered into Section IV. In Section IV, general information about lifestyle parameters, such as dietary intake, caffeine, dairy consumption, water intake, stress, etc., can be retrieved.

**Figure 1:**
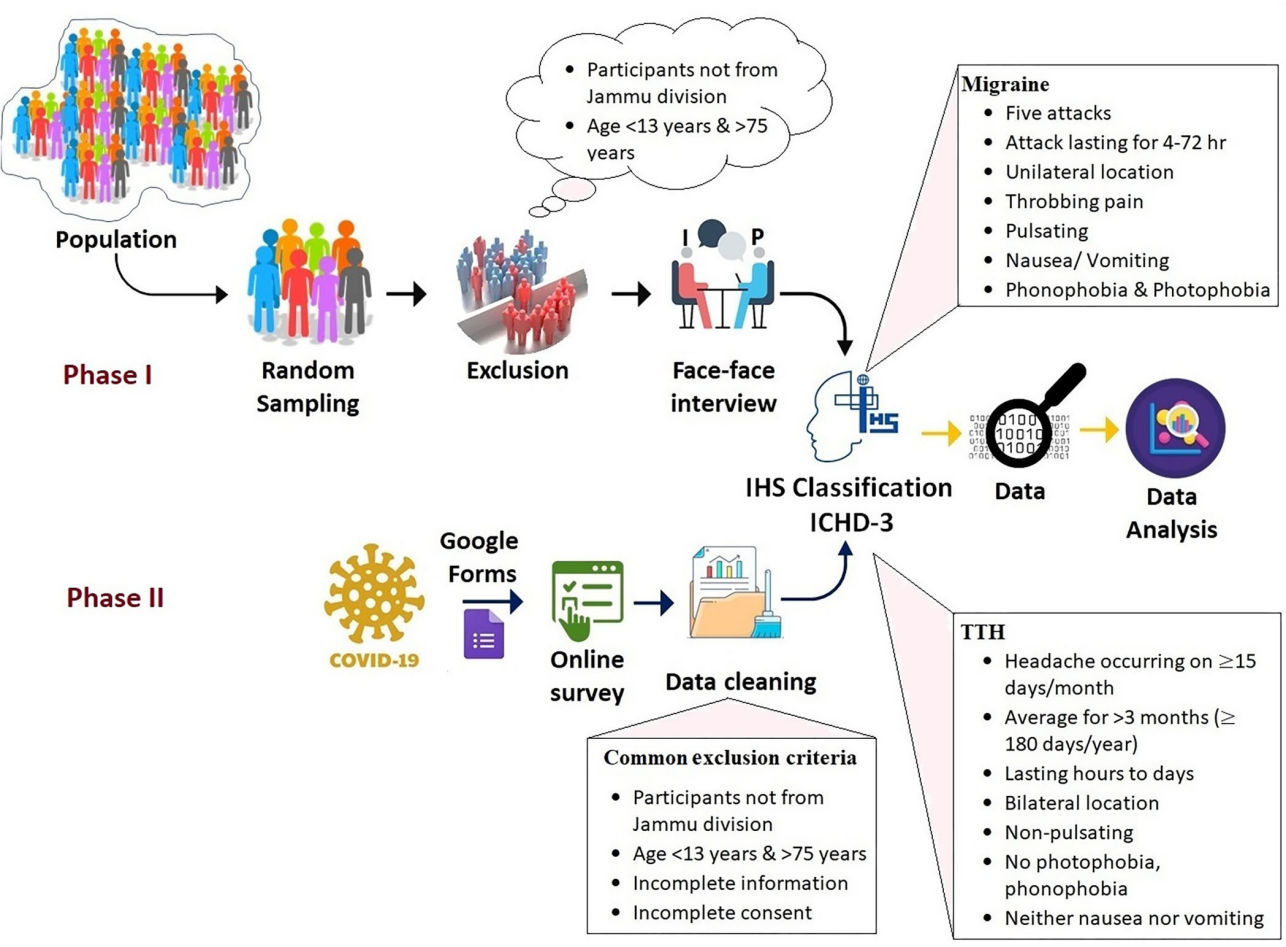
Pictorial representation of the data collection in the present survey.

After the collection of data utilizing the E-based method, the data were first cleaned using the exclusion criteria [such as: (1) Participants who have not given consent; (2) subjects with age <13 and >75; (3) subject data with partial/maximum blank information; (4) participants who were not from the Jammu division; and (5) duplicate data (utilizing “sort by feature method”)] presented in (Table 2) and then the cleaned data were merged with the data collected from the face-to-face interview. To diagnose different forms of headaches such as migraine, at least five attacks fulfilling the ICHD-3 criteria are presented in Table 1, which include the cardinal features such as unilateral location with throbbing/pulsating pain nature, along with nausea, vomiting, and importantly the phono- and photophobia. For TTH, the subject must reply to three of the following four features that include bilateral location of pain, with non-pulsating quality with mild intensity, no phono- and photophobia, and menstrually related migraine (Table 1). The strategy or schematic concept for the present study is depicted in the flow diagram (Figure 1).

**Table 2:** Exclusion criteria.

**Exclusion criteria**
1. Participants who have not given consent were initially excluded
2.Subjects with age <13 and >75 were excluded
3.Subject data with partial/maximum blank information were excluded
4.Participants not from the Jammu division were excluded
5. Duplicate data (utilizing “sort by feature method”) were also excluded

The sample size calculation was done to find out the statistical power utilizing “OpenEpi sample size for a proportion or descriptive study calculator” using a population size of 6,051,329 (Jammu and Kashmir Population 2022 (Indiacensus.net)] with previously published headache prevalence (11) and found significant power, i.e., >80%.

### 2.2. Statement of ethics

The present study was performed under the norms of the Institutional Ethical Committee (ICE) and guidelines of the Medical Council of India and was duly approved by the IEC of the University of Jammu vide notification number EC: DRS/22/4969 and by the IEC of the Government Medical College, Jammu, India (ECR/454/Inst/JK/2013/RR-20). Data collection was done only after receiving informed written consent from each study participant.

### 2.3. Statistical analysis

For the descriptive data analysis of continuous and discrete variables, mean ± standard deviation and frequency distribution methods, respectively, were utilized. To find out the significant difference between the continuous variable and discrete variables, *t*-test and chi-square analysis, respectively, were used. Adjusted odds ratio (AOR) using multiple logistic regression (LR) was used to find out the association between the dependent (headache and its type) and independent variables. All calculations were done using the free online statistical software including the *T*-test calculator (Graphpad.com), chi-square calculator [2 × 2–5 × 5 (Socscistatistics.com)], and MedCalc's statistical software for LR and 95% confidence interval (CI), and the *p*-value <0.05 was considered as a significant threshold for all statistical tests.

The prevalence of factors associated with the condition was observed using the “prevalence ratio” (PR) (13, 14). Due to the high prevalence of migraine worldwide (2), the PR was obtained utilizing the formula given in Equation 1:


(1) $$\begin{array}{l} PR\;=\;\left(N_{D}^{E}/N_{E}\right)/\left(N_{D}^{U}/N_{U}\right) \end{array}$$

wherein “PR” represents the “prevalence ratio,” “$N_{D}^{E}$” represents the “number of exposed subjects have a disease,” and “*N*_*E*_” is the “total number of individuals in the exposed group”. “$N_{D}^{U}$” shows the “number of subjects which are un-exposed but have the disease” and “*N*_*U*_” represents the total “number of that are unexposed subjects.”

To establish the prevalence difference (PD), i.e., “How many more cases are present in the exposed group than the unexposed group?”, Equation 2 was used, where “PD” represents the “prevalence difference (expressed in prevalent cases in exposed per 100 compared to the unexposed group).” All inputs were done in Microsoft Excel-2019.


(2) $$\begin{array}{l} PD\;=\;\left(\frac{N_{D}^{E}}{N_{E}}\right)-\left(\frac{N_{D}^{U}}{N_{U}}\right). \end{array}$$

## 3. Result

### 3.1. Demographic characteristics

**Figure 2:**
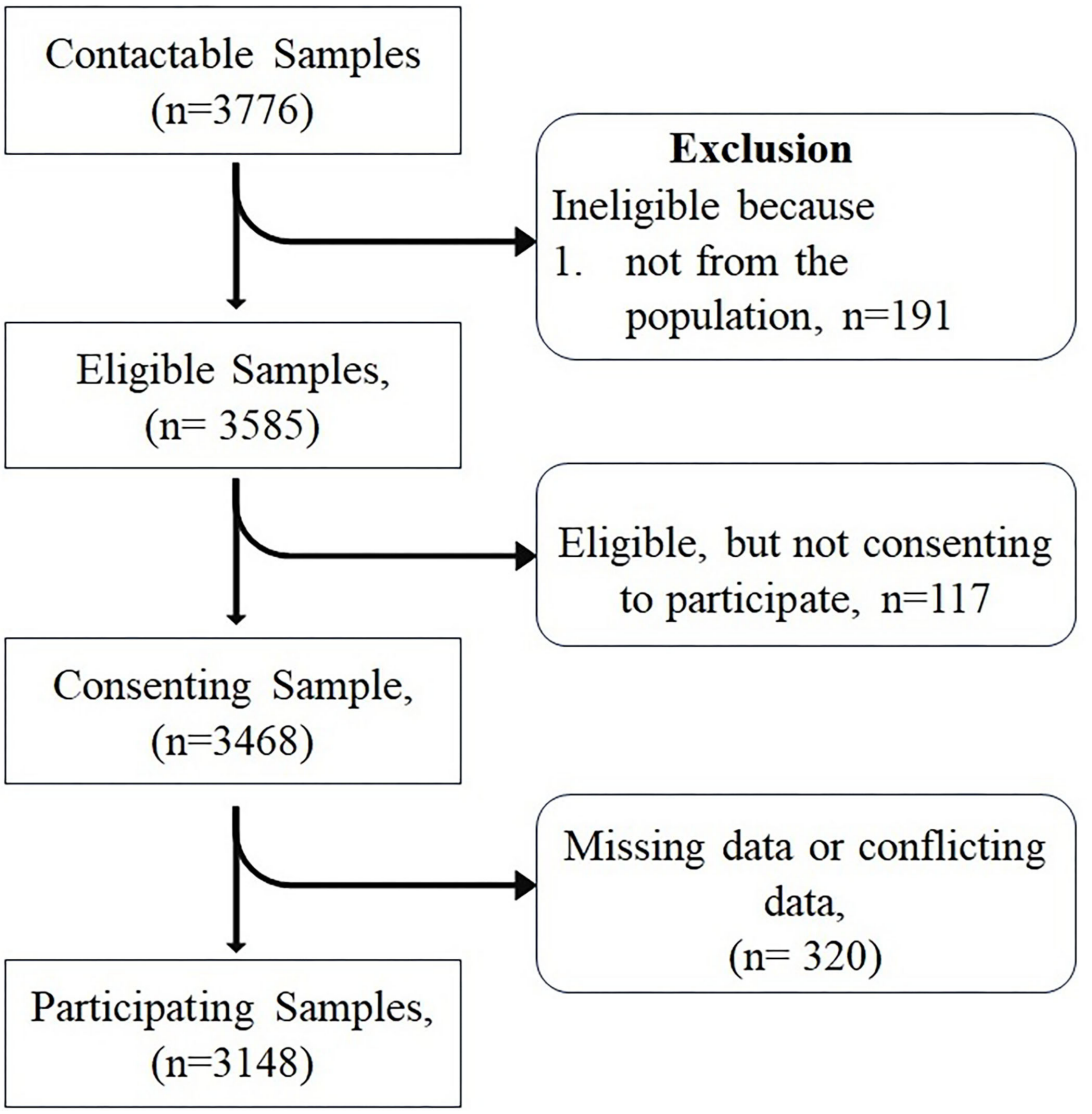
Flowchart of participation.

**Figure 3:**
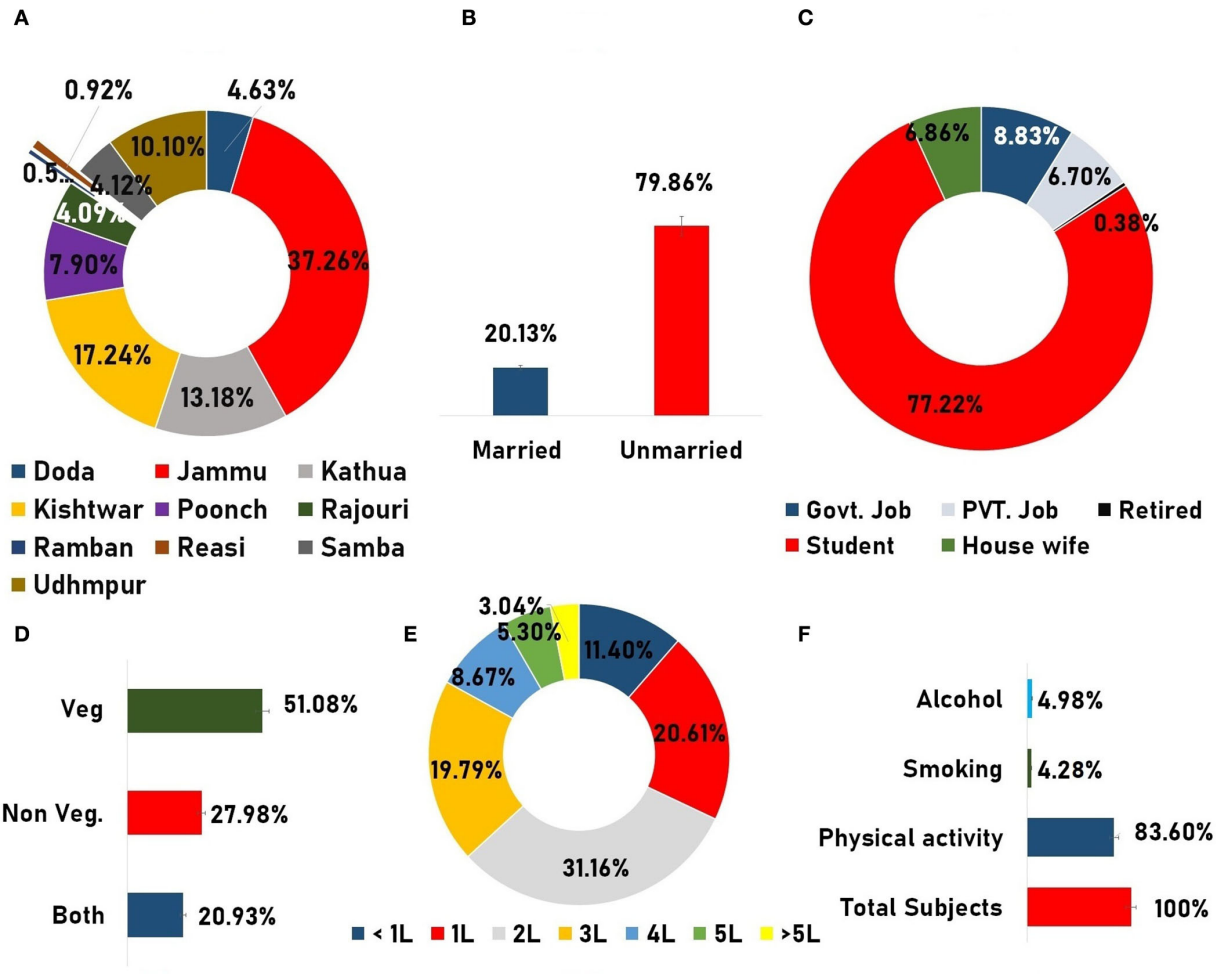
Demographic infographics. **(A)** Doughnut graph representing the total subject inclusion from different parts of the Jammu division. **(B)** Column graph showing the marital status. **(C)** Doughnut graph representing the occupation status of the participants. **(D)** Dietary habit of the participants. **(E)** Doughnut diagram showing the frequency of the total water intake in liters by the participants. **(F)** Presentation of frequency of different data variables such as “physical activities,” “smoking,” andand “alcohol usage”.

A total of 3,585 subjects were found to be initially eligible for the present study, but after utilizing the stepwise exclusion, only 3,148 subjects were included, and therefore the participation rate was found to be quite good (participation vs. eligible: participation rate 87.8%; Figure 2). Finally, a total of 3,148 subjects (24.87 ± 10.32) representing the male 1,223 (38.85%) and female 1,925 (61.14%) with mean ages of 25.73 ± 11.55 and 24.32 ± 9.42, respectively, were included. The difference between the mean ages was found to be extremely statistically significant (*p*-value = 0.0002). The participants who were included came from different regions of the Jammu Division, where many of them were seen to hail from the Jammu district (37.26%), Kishtwar (17.24%), Kathua (13.18%), and Udhampur (10.10%) districts (Figure 3A). As regards the marital status of the participants, the majority of them were unmarried (79.86%; Figure 3B) and as regards the occupation status of the participants, the majority of them were found to be students (77.22%; Figure 3C). It seems that the individuals mostly favor the vegetarian diet (51.08%) followed by individuals who prefer the non-vegetarian diet (27.98%; Figure 3D). The intake of water (measured in liters) was also observed, wherein it was found that the majority of the participants (31.16 %) drink 2 L of water per day in contrast with the least, i.e., 3.04% who drink more than 5 L/day (Figure 3E). Other lifestyle factor estimates, including smoking, alcohol, and physical activity, are presented in (Figure 3F). The detailed demographic features (such as age, marital status, occupation, community, water intake, diet, caffeine, diary product, junk food, smoking, and alcohol) and frequency distribution of the participants are presented in (Table 3).

**Table 3:** Frequency distribution of demographic features.

**Variable**	**Grouping**	**Total (*n*) (%)**	**Male (*n*) (%)**	**Female (*n*) (%)**
Sample size	N/A	3,148	1,223	1,925
Calculation	N/A	*n*/3,148*100	*n*/1,223*100	*n*/1,925*100
Age	10–19 years	1,072 (34.05%)	448 (36.63%)	624 (32.41%)
	20–35 years	1,656 (52.60%)	580 (47.42%)	1,076 (55.89%)
	36–55 years	337 (10.70%)	148 (12.10%)	189 (9.81%)
	56–75 years.	84 (2.66%)	47 (3.84%)	37 (1.92%)
Marital status	Married	633 (20.10%)	274 (22.40%)	359 (18.64%)
	Unmarried	2,514 (79.86%)	948 (77.51%)	1,566 (81.35%)
	Divorce	1 (0.03%)	1 (0.08%)	0 (0%)
Occupation	Student	2,431 (77.22%)	889 (72.69%)	1,542 (80.10%)
	Govt. job	278 (8.83%)	166 (13.57%)	112 (5.81%)
	Pvt. job	211 (6.70%)	156 (12.75%)	55 (2.85%)
	Retired	12 (0.38 %)	12 (0.98%)	0 (0%)
	House wife	216 (6.86%)	0 (0%)	216 (11.22%)
Community	Hindu	2,273 (72.20%)	799 (65.33%)	1,474 (76.57%)
	Muslim	809 (25.69%)	405 (33.11%)	404 (20.98%)
	Sikh	61 (1.93%)	19 (1.55%)	42 (2.18%)
	Buddhist	5 (0.15%)	0 (0%)	5 (0.25%)
Water intake	<1 L	359 (11.40%)	87 (7.11%)	272 (14.12%)
	1 L	649 (20.61%)	218 (1.78%)	431 (22.38%)
	2 L	981 (31.16%)	397 (32.46%)	584 (30.33%)
	3 L	623 (19.79%)	260 (21.25%)	363 (18.85%)
	4 L	273 (8.67%)	129 (10.54%)	144 (7.48%)
	5 L	167 (5.30%)	83 (6.78%)	84 (4.36%)
	>5 L	96 (3.04%)	49 (4.006%)	47 (2.44%)
Diet	Veg	1,608 (51.08%)	486 (39.73%)	1,122 (58.28%)
	Non-veg	881 (27.98%)	406 (33.19%)	475 (24.67%)
	Both	659 (20.93%)	331 (27.06%)	328 (17.03%)
Caffeine	N/A	2,237 (71.06%)	855 (69.91%)	1,382 (71.79%)
Dairy product	N/A	2,547 (80.90%)	951 (77.75%)	1,596 (82.90%)
Junk food	N/A	1,546 (49.11%)	542 (44.31%)	1,004 (52.15%)
Smoking	N/A	135 (4.28%)	115 (9.40%)	20 (1.03%)
Alcohol	N/A	157 (4.98%)	135 (11.03%)	22 (1.14%)

### 3.2. Prevalence

**Figure 4:**
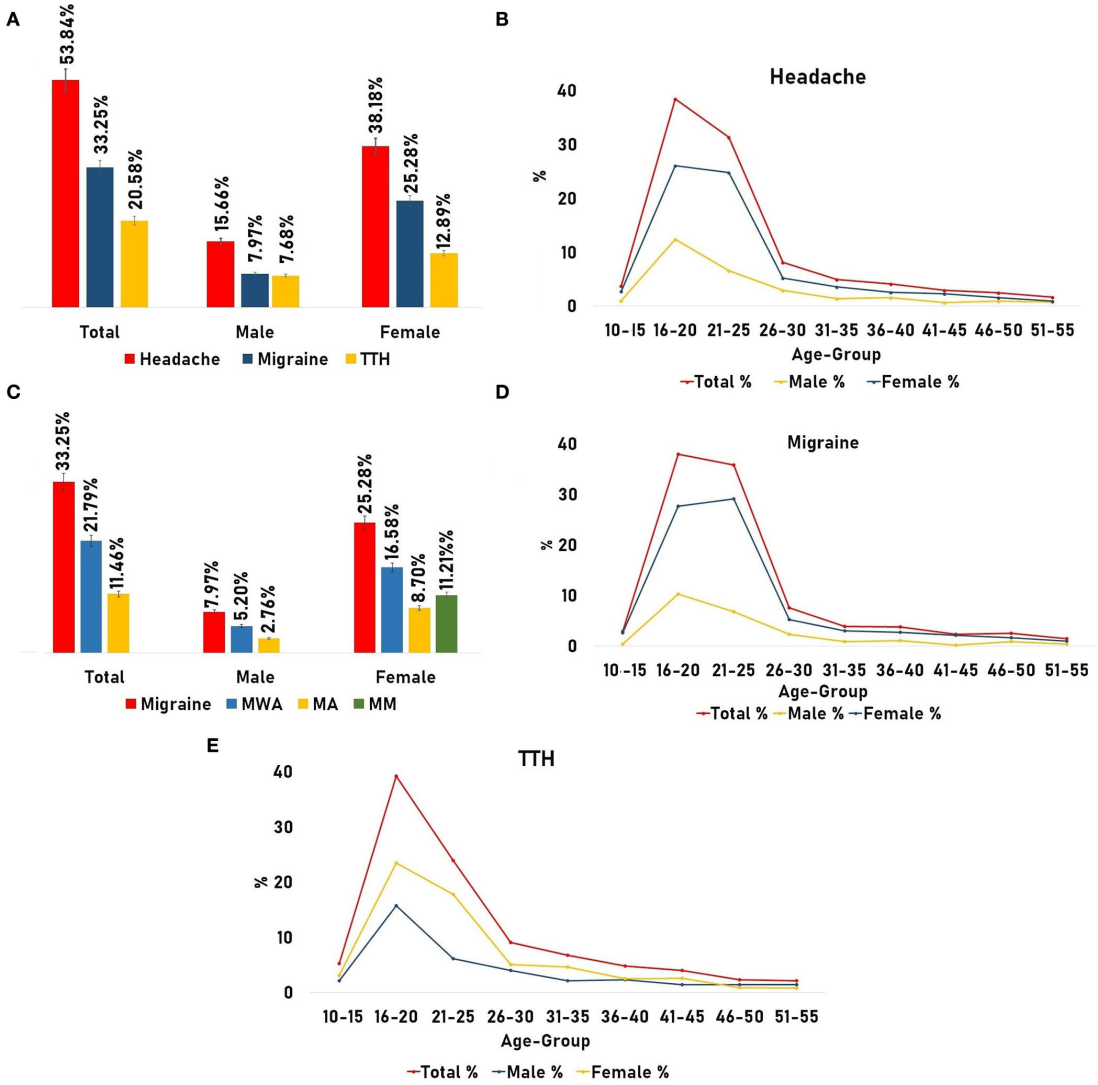
Prevalence of headache and its types. **(A)** Prevalence of headache with migraine and tension-type headaches (TTH) adjusted for gender. **(B)** Age and gender-adjusted prevalence of headache. **(C)** Prevalence of migraine and its type [Migraine Without Aura (MWA) and Migraine with Aura (MA)] adjusted for gender. **(D)** Age and gender-adjusted prevalence of migraine. **(E)** Age and gender-adjusted prevalence of TTH.

**Figure 5:**
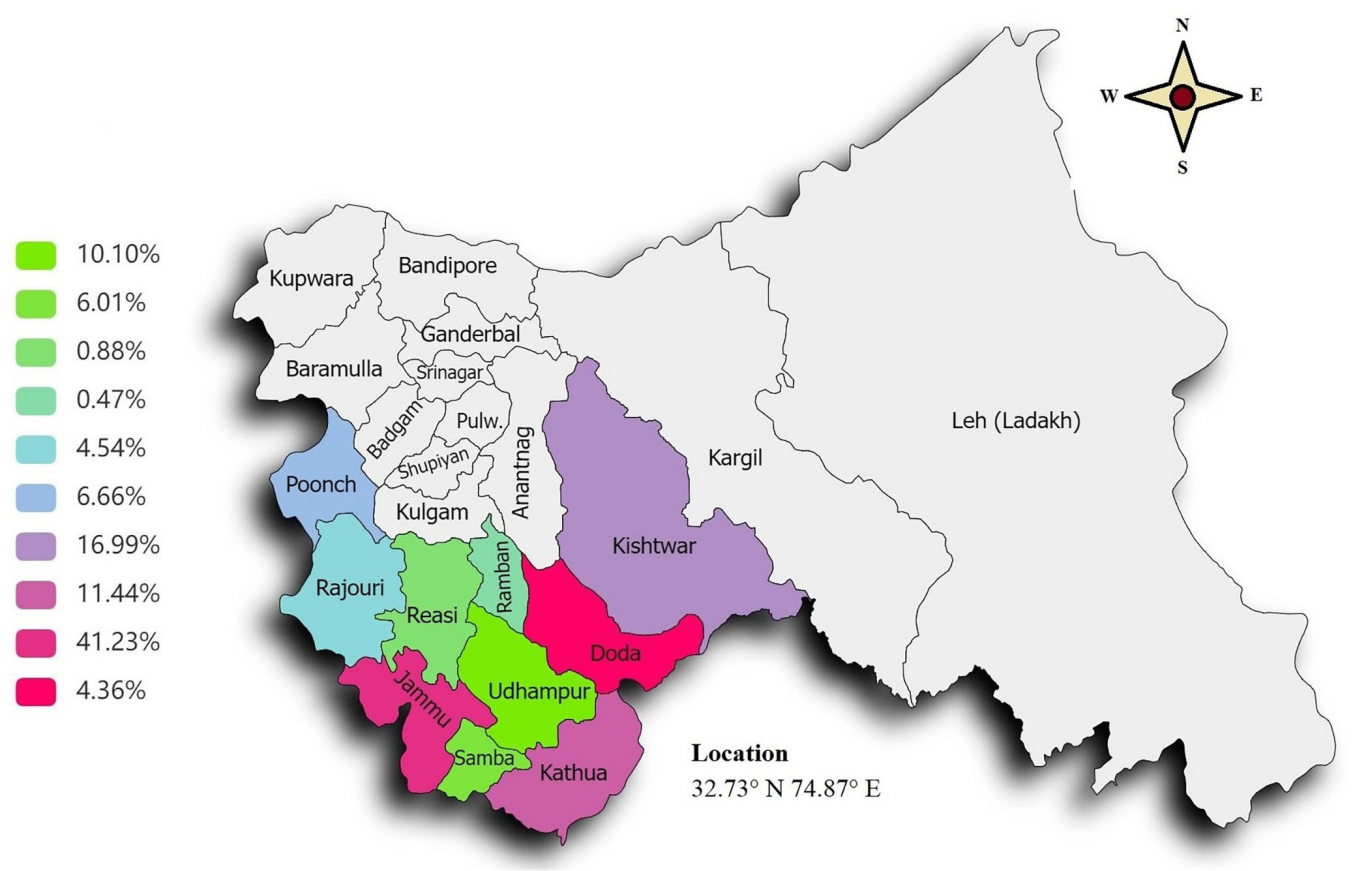
Prevalence of headaches in the Jammu region of the north Indian population.

In the present epidemiology study, the prevalence rate of headache was found to be near 53.84% (52.09–55.58; *n* = 1,695) with a mean age of 24.95 ± 10.06. Representing the total prevalence of males with a mean age of 25.83 ± 11.51, it was nearly 15.66% (14.39–16.92; *n* = 493) in comparison to females (mean age of 24.59 ± 9.39) where the rate of prevalence was quite high at 38.18% (36.48–39.88; *n* = 1,202; Figure 4A). A high rate of prevalence was observed in the age group of 15–26 years (Figure 4B). After sub-grouping of the “headache diagnosed participants” based on the criterion “where did they belong to?”, the maximum prevalence was observed in the Jammu district (41.23%), followed by the Kishtwar (16.99%), Kathua (11.44%), and Udhampur districts (10.10%). The frequency distribution of headache prevalence is presented in the Jammu & Kashmir map (Figure 5).

Using the ICHD-3 criteria (Table 1), different types of headaches were also observed, where the majority of the headache sufferers had to tolerate severe pain from the migraine type “a neurovascular inflammatory disorder,” which was nearly 33.25% (31.60–34.89; *n* = 1,047) including males (24.45 ± 10.76) representing the total prevalence of 7.97% (7.02–8.91; *n* = 251) and females (24.23 ± 9) near 25.28% (23.76–26.79; *n* = 796). After further sub-grouping of migraine, we observed that 21.79% (20.34–23.23) of migraine sufferers were Migraine Without Aura (MWA; *n* = 686), wherein females showed preponderance [16.58% (15.28–17.87; *n* = 522)] in contrast to males with 5.20% (4.42–5.97; *n* = 164; Figure 4C). Another migraine type, i.e., Migraine with Aura (MA), was found in nearly 11.46% (10.34–12.57; *n* = 361) of the total sufferers where females show preponderance with 8.70% (7.71–9.68; *n* = 274) in comparison to males [2.76%, (2.18–3.33; *n* = 87); Figure 4C]. It has been found that 11.21% (CI; *n* = 353) of female migraine sufferers had to tolerate pain from menstrual migraine. Also, another headache type, i.e., TTH was observed, where the total prevalence was found to be near 20.58% (19.16–21.99; *n* = 648), with females representing 12.89% (11.71–14.06; *n* = 406) and males 7.68% (6.74–8.61; *n* = 242; Figure 4A).

Grouping headache subjects on the basis of the “age group/age-adjusted prevalence,” it was observed that the highest prevalence was found in the age group of 20–35 years (mean age: 23.79±4.03; young adults; Figure 4B) representing 55.28% (52.98–57.58) with female dominance, i.e., 75.77% (73.00–78.54; male: 24.22%; Table 4). Migraine was found to be more prevalent (58.07%, 55.08–61.05) in the same age group (20–35 years) than TTH, i.e., 50.77% (46.92–54.61; Figures 4D, E). But in the middle-aged group (36–55 years), the prevalence of TTH was slightly increased by 3.27% (10.65–15.88) than migraine (9.83%; Table 4).

**Table 4:** One-year prevalence of headache and its type adjusted for age and gender.

**Age group**	**Mean**±**SD**		**Headache**			**Migraine**			**TTH**		
			**(*n* = 1,695)**	**(%)**	**95% CI**	**(*n* = 1,047)**	**(%)**	**95% CI**	**(*n* = 648)**	**%**	**95% CI**
14–19 (Adolescent)	17.65 ± 10.33	Total	532	31.38%	29.17–33.58	315	30.08%	27.30–32.85	217	33.48%	29.84–37.11
		Male	184	34.58%	30.56–38.6	88	27.93%	22.97–32.88	96	44.23%	37.62–50.83
		Female	348	65.41%	61.37–69.45	227	72.06%	67.10–77.01	121	55.76%	49.15–62.36
20–35 (YA)	23.79 ± 10.33	Total	937	55.28%	52.98–57.58	608	58.07%	55.081–61.05	329	50.77%	46.92–54.61
		Male	227	24.22%	17.95-30.49	127	20.88%	17.64–24.11	100	30.39%	25.41–35.36
		Female	710	75.77%	73–78.54	481	79.11%	75.87–82.34	229	69.60%	64.62–74.57
36–55 (MAG)	44.16 ± 10.35	Total	189	11.15%	9.66–12.64	103	9.83%	8.02–11.63	86	13.27%	10.65–15.88
		Male	65	34.39%	27.62–41.15	26	25.24%	16.85–33.629	39	45.34%	34.81–55.86
		Female	124	65.60%	58.83–72.37	77	74.75%	66.36–83.13	47	54.65%	44.12–65.17
56–75 (OAA)	61.21 ± 10.39	Total	37	2.18%	1.485–2.875	21	2.00%	1.15–2.84	16	2.46%	1.265–3.65
		Male	17	45.94%	29.88–61.99	10	47.61%	26.24–68.97	7	43.75%	19.44–68.05
		Female	20	54.05%	38–70.1	11	52.38%	31.01–73.74	9	56.25%	31.94–80.55
All			1,695	53.84%	52.09–55.58	1,047	33.25	31.60–34.89	648	20.58	19.16–21.99

YA, young adults; MAG, middle-aged group; OAA, old age group.

As regards the frequency of headaches, i.e., the number of attacks per month, the highest frequency with 7–14 attacks/month was observed in 41% of headache individuals and after analyzing the headache type, the maximum attack frequency was observed in migraine patients in comparison to TTH. Females were observed to show increased attack frequency per month in contrast to male subjects (Table 5). As regards the periodicity or pain initiation, no specific periodicity was observed and the majority of headache subjects showed that the pain initiates at any time of the day (Supplementary file).

**Table 5:** One-year prevalence of headache and its type on the number of attacks per month by gender.

	**Gender**	**1–7 attack/month**			**7–14 attack/month**			>**14 attack/month**		
		***N***	**%**	**95% CI**	***N***	**%**	**95% CI**	***N***	**%**	**95% CI**
Headache (1,695)	Total	535	31.56%	29.34–33.77	703	41.47%	39.12–43.81	457	26.96%	24.84–29.07
	Male	143	26.72%	22.96–30.47	226	32.14%	28.68–35.59	124	27.13%	23.05–31.20
	Female	392	73.27%	69.51–77.02	477	67.85%	64.39–71.30	333	72.86%	68.78–76.93
Migraine (1,047)	Total	338	32.28%	31.20–33.35	425	40.59%	37.61–43.56	284	27.12%	24.43–29.81
	Male	73	21.59%	17.20–25.97	114	26.82%	22.60–31.03	64	22.53%	17.67–27.38
	Female	265	78.40%	74.01–82.78	311	73.17%	68.95–77.38	220	77.46%	72.60–82.31
TTH (648)	Total	197	30.40%	26.85–33.94	278	42.90%	39.08–46.71	173	26.69%	23.28–30.09
	Male	70	35.53%	28.84–42.21	112	40.28%	34.51–46.04	60	34.68%	27.58–41.77
	Female	127	64.46%	57.77–71.14	166	59.71%	53.94–65.47	113	65.31%	58.21–72.40

### 3.3. Association

Multiple logistic regression was used to find out the association (adjusted odds ratio-AOR) between sociodemography, such as age (13–35 years and reference ≥36 years), gender female (reference male), and marital status (unmarried as reference; independent variables) and headache (dependent variable) and the estimates are given in Table 6.

**Table 6:** Multivariate logistic regression analysis for association with sociodemographic variable.

**Variable**	**Headache**			**Migraine**			**TTH**		
	**AOR**	**95% CI**	***p*-Value**	**AOR**	**95% CI**	***p*-Value**	**AOR**	**95% CI**	***p*-Value**
Age	1.3708	0.9882–1.9014	0.0589	1.2051	0.8151–1.7817	0.3496	1.6379	1.1099–2.4169	0.0129
Gender	2.4654	2.1275–2.8570	<0.0001	3.1916	2.6776–3.8042	<0.0001	1.7142	1.4161–2.0750	<0.0001
Married	1.4634	1.1154–1.9200	0.006	1.1355	0.8208–1.5709	0.4429	2.089	1.5211–2.8690	<0.0001

It is found that gender [female: AOR = 2.46, 95% CI (2.12–2.87)] and marital status [AOR: 1.46, 95% CI (1.11–1.92)] show a significant association with headaches in contrast to age. As regards headache types such as migraine and headache, a significant association was found between all independent variables under study in contrast to the migraine group (Table 6).

### 3.4. Prevalence ratio and prevalence difference

To analyze the PR, first, a significant difference in the frequency distribution of factors between the headache with different types of headaches and non-headache subjects was observed (Supplementary file). The difference in smoking (*p*-value = 0.46) and alcohol consumption (*p*-value = 0.57) was not found to be significant between headache and non-headache individuals. But after stratification/sub-grouping, a significant difference (*p*-value = 0.05) was observed between the two groups' subjects in contrast to TTH (*p*-value = 0.31). In addition, statistically significant differences were found between the headache/headache type and non-headache for different lifestyle factors (Supplementary file).

**Figure 6:**
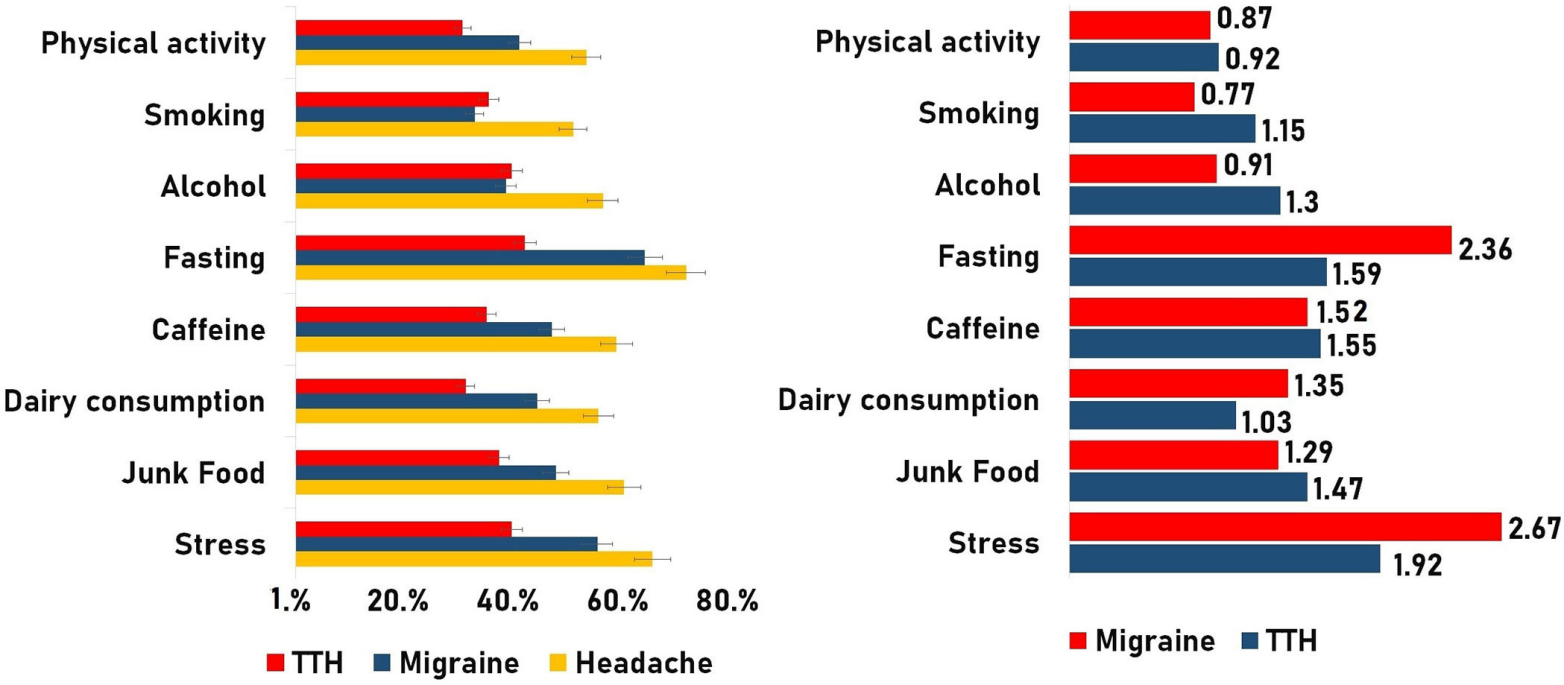
Two-dimensional (2D) bar graph representing. **(Left)** Prevalence of diseases under different exposures, **(Right)** Prevalence ratio in migraine and tension-type headaches (TTH).

After comparing and finding out the significant differences, the risk attribution of different lifestyle factors was evaluated using PR (14). Such an effect of different lifestyle factors on the prevalence of headaches was evaluated by PR utilizing Equation 1. This resulted in the identification of various environmental exposures (Table 7), which were found to be responsible for significant increase in the prevalence of the condition (Figure 6). Such factors include fasting, stress, consumption of junk food, dairy food consumption, caffeine intake, etc.

**Table 7:** Prevalence ratio of outcome under different lifestyle exposures/independent variables.

**Type**	**Variable**	**Disease prevalence in exposed**	**Disease prevalence in non-exposed**	**Prevalence ratio**	**95% CI**	**Prevalence difference**
Headache	Stress	65.20%	34.10%	1.91	1.75–2.08	31.12
	Junk food	60.00%	47.90%	1.25	1.12–1.29	12.02
	Female (gender)	62.4%	40.3%	1.54	1.43–1.67	22.13
	Dairy consumption	55.30%	47.60%	1.16	1.06–1.27	7.73
	Caffeine	58.60%	42.20%	1.38	1.27–1.50	16.38
	Fasting	71.30%	42.00%	1.69	1.59–1.80	29.25
	Alcohol	56.10%	53.80%	1.04	0.90–1.20	2.29
	Smoking	50.70%	54.00%	0.94	0.79–1.11	−3.24
	Physical activity	53.10%	57.80%	0.91	0.84–0.99	−4.67
Migraine	Stress	55.10%	20.60%	2.67	2.34–3.04	34.45
	Junk food	47.50%	36.80%	1.29	1.17–1.41	10.72
	Female	52.4%	25.6%	2.04	1.82–2.30	12.09
	Dairy consumption	44.10%	32.40%	1.35	1.18–1.56	11.65
	Caffeine	46.8%	30.6%	1.52	1.35–1.72	4.37
	Fasting	63.80%	27%	2.36	2.14–2.59	36.8
	Alcohol	38.40%	42.10%	0.91	0.71–1.15	−3.67
	Smoking	32.70%	42.30%	0.77	0.57–1.03	−9.6
	Physical activity	40.9%	46.7%	0.87	0.78–0.98	−5.76
TTH	Stress	39.40%	20.50%	1.92	1.66–2.21	18.92
	Junk food	37.20%	25.30%	1.47	1.29–1.67	11.89
	Female	36%	24.9%	1.44	1.26–1.65	11.06
	Dairy consumption	31.10%	30%	1.03	0.88–1.21	1.07
	Caffeine	34.90%	22.4%	1.55	1.32–1.81	12.44
	Fasting	41.80%	26.20%	1.59	1.40–1.81	15.6
	Alcohol	39.50%	30.40%	1.3	1.02–1.64	9.11
	Smoking	35.30%	30.60%	1.15	0.87–1.51	4.67
	Physical activity	30.5%	32.90%	0.92	0.78–1.09	−2.46

As we have observed, the proportion of subjects with headaches is 1.9-fold greater in stressed subjects as compared to unstressed subjects. As regards this scenario, there were 31.1 excess cases of headache per 100 in the stress group compared to the non-stress individuals in a specific time. After facing stress, different lifestyle factors were also found to increase the proportion of conditions that include dairy consumption (1.16-fold), junk food (1.25-fold), caffeine consumption (1.38-fold), fasting (1.69-fold), etc. (Table 7). A comparison of the prevalence ratio in migraine and TTH has also been explored (Figure 6).

It is important to note that, smoking and physical activity showed a negative prevalence difference in headache but after sub-grouping into types, smoking showed a profound effect on the prevalence of TTH increasing by 1.15-fold, as compared to migraine (PR:0.77; Figure 6), and representing an excess of 4.67 cases of TTH per 100 compared to non-smokers. Also, the effect of alcohol consumption was turned down by the migraine group (PD: −3.67) in comparison to TTH (PR: 1.3-fold and PD: 9.11 cases/100; Figure 6).

Enclosing the section, based on the current cross-sectional study, different lifestyle parameters have a profound effect on the prevalence of headaches and their different forms (migraine and TTH).

## 4. Discussion

### 4.1. Prevalence rate

The GBD-2019 has revealed that the prevalence of headaches is much higher in the top economically developed nations, with the greatest prevalence rates being observed in Italy (49.02%) and the USA (45.11%). It has been discovered that 15.8% of people worldwide experience headaches everyday (15). As regards India's neighboring countries, the highest prevalence was recorded in Sri Lanka (37.2%) followed by Nepal (35.95), and also in the states of India, which include Sikkim (38.08%) and Goa (35.24%) (2).

In the current study, estimates of high headache prevalence were found (Figure 4), which are also consistent with those of other epidemiological studies conducted in different regions of India. In south India, the prevalence of headache stands at 63.9% with a female preponderance of 73% in comparison to males (54.4%), TTH is 34.8%, and migraine 25.6% (females: 32.4% and males: 18.6%) (8). In the eastern states of India, the prevalence of headache stands at 14.87% where the female preponderance amounts to 23.51% and that of males 5.44% and the percentage of migraine 14.12% (males: 5.35 and 22.16%) (9). In the north Indian region, different studies have shown that the prevalence of headache is 63.9%, wherein females were found to be affected more (74.3%) as compared to males (32.6%). The prevalence of migraine was 13.44% with a female preponderance (87.5%) (16). A prospective observational study on the north Indian population found that 67.7% of patients had migraine and 32.2% of patients had TTH (17). In the Kashmir Valley, headache in the pediatric population was observed where the headache frequency rate was found to be at 66.4%, with the females at 65.15% and males at 35.85%, migraine at 26.98%, and TTH at 50.99% (10). Another group found the headache frequency rate to be equal to 66.20% (19–45 years) with female dominance (61.82 %) to male (38.18 %). In migraine, the total prevalence was found to be 45.69%, with 55.44 % in females and 32.0 % in males (11).

Interestingly, the current study was also found to be consistent with other epidemiological studies conducted outside of India, including the cross-sectional community-based research on the Kuwaiti population, which have indicated a high incidence of headache (61.37%) and type of headache, with TTH being the prevalent type (29.06%) in contrast to migraine (23%) (18, 19), in Pakistani population with high prevalence of headaches (76.6%; TTH: 44.7% and migraine 22.9%) wherein female migraineurs showed a preponderance (26.9%) in contrast to male TTH (51.2%) (20). A meta-analysis of 302 community-based studies with a total sample size of 6,216,995 revealed that Central and South America have the greatest migraine prevalence (16.4%) followed by Europe (11.4%), Africa (10.4%), Asian countries (10.1%), and North America (9.7%) (21). A recent GBD meta-update showed the rising prevalence of migraine over time in contrast to other headache types (15).

To this end, the picture emerging from the current study is of a high proportion of people suffering from headaches with different forms such as migraine, MM, and TTH. It is noteworthy to understand that the prevalence of headache and its type varies from region to region and this disparity is quite huge due to the different sampling approaches (simple random, clustered, stratified sampling), different sample sizes, type of study (population-based/hospital case-control, cohort), the differing methodology adopted, differences in defining the criteria of headache prevalence (1 year vs. 3 months), coexisting environmental factors, urban/rural differences, or ethnicity of the studied population.

### 4.2. Prevalence ratio

In the current descriptive cross-sectional study design, the prevalence of factors associated with the condition was observed using the “prevalence ratio” (13). To the best of our knowledge, this is the first study on headache epidemiology that has utilized PR as a measure of association other than the odds ratio in a cross-sectional study design (22). It was found that in the stress group, the prevalence of headaches was high (65%; Figure 6), and it is well known that stress has a major influence on headache, and its frequency also when the latter is directly proportional to stress intensity (23, 24). Different forms include occupational stress (25–27) and educational stress (28, 29) which have all been found to be associated with headaches.

The current study provides insight into the prevalence-risk potential of caffeine intake (PR: 1.38; Figure 6), and in support of our study, a population-based case-control study has found that patients with Chronic Daily Headache (CDH) were more likely overall to have been high caffeine consumers before the onset of CDH (30). Also, a prospective cohort study, conducted by Mostofsky et al. (31), found that high levels of caffeine beverage intake may be one of the triggers of migraine type. With such a study delving at length into the association of positive risks, it was also observed that sudden cessation of caffeine leads to a withdrawal syndrome with headache as the dominant symptom (32) and constipation, hand tremors, increased diuresis, and abdominal pain (33, 34) as additional symptoms.

In addition to caffeine intake, empty stomach/fasting significantly increases the prevalence of headache (71.30%) with migraine at the front of the head (63.82% and PR: 2.36) in comparison to TTH (41.80% and PR: 1.59; Figure 6). Such “Fasting-Induced Headache (FIH)” is dependent on the duration of the fast, and pain is featured as non-pulsating, mild to moderately intense, wide, and centered in the unilateral/frontal area (35). Also, if an individual takes a meal after a long duration of fasting, the chance of “postprandial fasting-related headaches” increases which are featured with episodic pain, and heaviness (36). In addition to fasting, the consumption of fast-food and dairy products (such as milk, curd, ice creams, etc.) significantly increases the prevalence rate (Figure 6). In support of our study, it was well-established that patients with migraines consumed less milk than patients without migraines (37, 38).

As regards the prevalence ratio of smoking, the prevalence of headache was low (50.70%) in the smoking group as compared to non-smokers (54%). But after sub-grouping, it was observed that people with TTH are 1.15-fold greater than people who do not smoke. Also, it was observed that the prevalence of migraine significantly drops in the alcohol group (38.40%) in contrast to the non-alcoholic group (42.10%; Table 7). The importance of alcohol as a migraine trigger did not show any justification but it has shown that low dose of alcohol can have a beneficial effect on migraine (39). Other than alcohol, smoking is unlikely to be a factor responsible for the cause or exacerbation of migraine due to the low prevalence of smokers with headaches (Table 7).

Enclosing the section, the present headache epidemiology study has presented a various range of environmental risk attributes (Figure 6) that significantly increase the likelihood of headache. Patients with headaches need to be aware of the risk factors that contribute to their condition, so that they may avoid them and possibly reduce their headache frequency (Graphical abstract).

### 4.3. Strengths and limitations of the study

Our study's strengths include the fact that it is the first of its type (cross-sectional descriptive study) to evaluate the prevalence of primary headache disorders among various age groups in the Jammu [Jammu & Kashmir (UT)] population of north India. In addition to prevalence, we have also explored different lifestyle attributes that are found to be responsible for the increased prevalence of headaches and their major two types, i.e., migraine and TTH. Such information on the rising prevalence of primary headache disorders and lifestyle factors may aid in drawing up the best plans for patient care in the target area. Despite such advantages of the study, an important limitation might be recall bias, which is customary in most studies using a questionnaire. Apart from such astonishing information, the prevalence ratio was unable to establish the temporal associations that could be understood better through an analytical study.

## 5. Conclusion

Headache affects millions of individuals across the country and it is defined as a complicated neurovascular condition, not merely a simple discomfort. In conclusion, headache is found to be a highly prevalent condition among the people of Jammu (north Indian population). Notably, several risk factors contribute to a higher prevalence of the condition causing headache, which may be reduced by avoiding the risk factors. Therefore, there should be such programs in place where a person suffering from headache is made aware of the potential risk factors that contribute to aggravating the condition so that they can avoid them at the outset and perhaps minimize the frequency of their headaches.

## Data availability statement

The original contributions presented in the study are included in the article/Supplementary material, further inquiries can be directed to the corresponding author.

## Ethics statement

The studies involving human participants were reviewed and approved by Institutional Ethical Committee, University of Jammu and Government Medical College, Jammu, Jammu & Kashmir, India. Written informed consent to participate in this study was provided by the participants' legal guardian/next of kin.

## Author contributions

PK and AS contributed to the study design. HK, PK, MY, and AS conducted the survey. AP, SB, and SS helped with data cleaning. AP and AS analyzed the data. AS drafted the manuscript. AS, AP, and SS edited the pictures and table. PK, MY, and RP edited the manuscript. HK and PK finalized the manuscript. All authors contributed to the article and approved the submitted version.
