# Supplementary material for: Neuroepidemiology study of headache in the region of Jammu of north Indian population: A cross-sectional study
Source: Front Neurol. 2023 Jan 5;13:1030940. doi: 10.3389/fneur.2022.1030940 (PMC9850838; doi:10.3389/fneur.2022.1030940)
Supplement: Supplementary file 1 [file Data_Sheet_1.docx]

**Table 1:** Diversity in periodicity among headache and its different type

|  | Grouping | Morning (n) (%) | Afternoon (n) (%) | Evening (n) (%) | Anytime (n) (%) |
| --- | --- | --- | --- | --- | --- |
| Headache | Total | 129 (7.61%) | 257 (15.16%) | 155 (9.14%) | 1154 (68.08%) |
|  | Male | 50 (38.75%) | 76 (29.57%) | 51 (32.90%) | 316 (27.38%) |
|  | Female | 79 (61.24%) | 181 (70.42%) | 104 (67.09%) | 838 (72.61%) |
| Migraine | Total | 98 (9.3%) | 163 (15.56%) | 81 (7.73%) | 705 (67.33%) |
|  | Male | 38 (38.77%) | 51 (31.28%) | 20 (24.69%) | 142 (20.14%) |
|  | Female | 60 (61.22%) | 112 (68.71%) | 61 (75.30%) | 536 (76.02%) |
| TTH | Total | 31 (4.7%) | 94 (14.50%) | 74 (11.41%) | 449 (69.2%) |
|  | Male | 12 (38.70%) | 25 (26.59%) | 31 (41.89%) | 174 (38.75%) |
|  | Female | 19 (61.29%) | 69 (73.40%) | 43 (58.10%) | 275 (61.24%) |

**Table 2: Significant difference between dependent and independent variable**

| Variable | Response | Headache | No headache | X^2^ square | p-value | Migraine | X^2^ square | p-value | TTH | X^2^ square | p-value |
| --- | --- | --- | --- | --- | --- | --- | --- | --- | --- | --- | --- |
| Stress | Yes | 1302 | 694 | 284.56 | <0.00001 | 850 | 287.79 | <0.0001 | 452 | 87.4 | <0.0001 |
|  | No | 393 | 759 |  |  | 197 |  |  | 196 |  |  |
| Junk | Yes | 927 | 619 | 45.74 | <0.0001 | 561 | 29.43 | <0.0001 | 366 | 34.66 | <0.0001 |
|  | No | 768 | 834 |  |  | 486 |  |  | 282 |  |  |
| Dairy | Yes | 1409 | 1138 | 11.699 | =0.0006 | 896 | 21.13 | <0.0001 | 513 | 0.19 | 0.662 |
|  | No | 286 | 315 |  |  | 151 |  |  | 135 |  |  |
| Caffeine | Yes | 1311 | 927 | 69.85 | <0.0001 | 815 | 56.79 | <0.0001 | 496 | 33.3 | <0.0001 |
|  | No | 384 | 526 |  |  | 232 |  |  | 152 |  |  |
| PA | Yes | 1397 | 1235 | 3.79 | =0.05 | 856 | 4.66 | 0.03 | 541 | 0.78 | 0.37 |
|  | No | 298 | 218 |  |  | 191 |  |  | 107 |  |  |
| Fasting | Yes | 906 | 365 | 260.84 | <0.0001 | 644 | 334.74 | <0.0001 | 262 | 50.18 | <0.0001 |
|  | No | 789 | 1088 |  |  | 403 |  |  | 386 |  |  |
| Smoking | Yes | 68 | 66 | 0.5 | =0.46 | 32 | 3.5 | 0.05 | 36 | 0.9 | 0.31 |
|  | No | 1627 | 1387 |  |  | 1015 |  |  | 612 |  |  |
| Alcohol | Yes | 88 | 69 | 0.3 | =0.57 | 43 | 0.59 | 0.44 | 45 | 4.19 | 0.04 |
|  | No | 1608 | 1383 |  |  | 1004 |  |  | 603 |  |  |

**PA:** Physical activity**, Res.:** Response
